# Neuroprotective Effect of Ketone Metabolism on Inhibiting Inflammatory Response by Regulating Macrophage Polarization After Acute Cervical Spinal Cord Injury in Rats

**DOI:** 10.3389/fnins.2020.583611

**Published:** 2020-10-23

**Authors:** Junyu Lin, Zucheng Huang, Junhao Liu, Zhiping Huang, Yapu Liu, Qi Liu, Zhou Yang, Ruoyao Li, Xiuhua Wu, Zhe Shi, Qingan Zhu, Xiaoliang Wu

**Affiliations:** Division of Spinal Surgery, Department of Orthopaedics, Nanfang Hospital, Southern Medical University, Guangzhou, China

**Keywords:** spinal cord injury, ketone metabolism, ketogenic diet, inflammation, macrophage

## Abstract

**Objective:**

To investigate the effects of ketogenic metabolism on macrophage polarization, inflammation inhibition, and function recovery after acute spinal cord injury (SCI) in rats.

**Methods:**

Sixty-four adult male Sprague–Dawley rats were randomly and equally divided into sham, standard diet (SD), ketone diet (KD), and 1, 3-butanediol (BD) groups. All animals underwent C5 unilateral laminectomy, whereas the SD, KD, and BD groups underwent C5 spinal cord hemi-contusion. The impact rod with a diameter of 1.5 mm was aligned 22.5° to the left and 1.4 mm to the midline, and then triggered to deliver a set displacement of 1.5 mm at a speed of 100 mm/s. The gene expression of inflammatory factors as well as the protein expression of inducible nitric oxide synthase, arginase-1, and inflammatory factors were measured at 1 week post-injury. Serum ketone and behavior were evaluated every second week for 12 weeks. Then, histological analyses of the gray and white matter at the epicenter were conducted at 12 weeks post-injury.

**Results:**

The serum ketone levels of the KD and BD groups were significantly increased when compared with the SD group. The gene and protein expression of TNF-α and IL-1β tended to increase after the SCI, but were inhibited in the KD and BD groups. The protein expression of inducible nitric oxide synthase, marker of M1 macrophage, was inhibited in the KD and BD groups; on the other hand, the expression of arginase-1, marker of M2 macrophage, was boosted in the KD and BD groups. The usage of the ipsilateral forelimb was higher in the KD group than in the SD group. The hemi-contusive injury resulted in an obvious ipsilateral lesion area at the epicenter, and there was no significant difference between groups regarding the lesion size. However, the spared gray matter area was significantly greater in the KD group than in the SD and BD groups.

**Conclusion:**

The present study suggests that ketogenic metabolism promotes macrophage polarization to M2, inhibits an inflammatory response, and alleviates the loss of gray matter after SCI. A higher ketone level, such as that induced by the ketogenic diet, seems to benefit function recovery after SCI.

## Introduction

Ketone bodies, including acetoacetate, acetone, and beta-hydroxybutyrate (βOHB), are generated in a state of starvation, fasting, or high-fat diet. Ketogenic metabolism, commonly induced by ketogenic food and calorie restriction, has been used as a therapeutic alternative because of its neuroprotective effect in neurological diseases. It was proven to be effective in the clinical treatment of pharmaco-resistant epilepsy, especially in children (Kverneland et al., 2017). Furthermore, ketogenic metabolism presented a beneficial effect in Alzheimer’s disease (Pawlosky et al., 2017), Parkinson’s disease (Norwitz et al., 2019), traumatic brain injury (Prins and Matsumoto, 2014; Wood et al., 2018), and amyotrophic lateral sclerosis (Zhao et al., 2006; Caplliure-Llopis et al., 2019).

In recent years, studies regarding the application of ketogenic metabolism in spinal cord injury (SCI) were reported. Previously, we investigated the tolerance of ketogenic diet (KD) and detected the serum ketone body levels in 10 patients with SCI in a clinical trial, thus confirming the safety and feasibility of KD (Guo et al., 2014). Yarar-Fisher et al. (2018) further proposed that KD has anti-inflammatory and neuroprotective effects, and could promote the recovery of neural function post-SCI. It was verified by Streijger et al. (2013) that KD was effective in promoting motor function, reducing the lesion area, and preserving greater spared gray matter after SCI in rats while Jeong et al. (2011) reported that fasting every other day, another form of ketogenic metabolism, was beneficial in protecting neural function and facilitating motor recovery. However, given the compliance of the patients, neither KD nor fasting every other day may be the best option, and may present challenges in clinical application because both require strict calorie restriction. Therefore, it is necessary to explore whether 1, 3-butanediol (BD), a ketone supplement that can promote the serum ketone level without interrupting normal diet (Kesl et al., 2016), possesses a neuroprotective effect and thus can offer a therapeutic alternative for SCI.

The pathophysiological mechanism of SCI is complex because an inflammatory response, oxidative stress, ischemic edema, hemorrhage, and hypoxia all occur in the secondary injury (Rogers and Todd, 2016). Our previous studies indicated that βOHB was the inhibitor of class I histone deacetylases and attenuated the oxidative stress post-SCI (Kong et al., 2017; Wang et al., 2017). Although, it was widely acknowledged that ketogenic metabolism acts through a combination of mechanisms (Boison, 2017), its role in regulating the inflammatory response, especially macrophage polarization, a crucial element of secondary pathology after SCI, remains to be clarified. It was reported that βOHB had an anti-inflammatory effect and inhibited the activation of NLRP3 inflammasome, which could accelerate the release of various pro-inflammatory cytokines (Youm et al., 2015). Activated macrophage would polarize into M1 and M2 sub-types and provided pro-inflammatory and anti-inflammatory effects, respectively, in terms of the inflammatory response after central nerve system injury (Kigerl et al., 2009). Recently, a study by Huang C. et al. (2018) showed that βOHB induced a functional ramification of murine microglia *in vitro* and *in vivo*, and that this ramification helped microglia to revert back to their tissue surveying and anti-inflammatory status in mice brain. However, we have been unable to locate any study conducted to evaluate the relation between ketogenic metabolism and macrophage polarization after SCI.

Therefore, the present study aims to investigate the neuroprotection of ketogenic metabolism, focusing on its effect on inhibiting an inflammatory response and regulating macrophage polarization post-SCI. Furthermore, this study investigates whether BD exerts a neuroprotective effect by regulating macrophage polarization.

## Materials and Methods

### Animal and Grouping

A total of 64 adult male Sprague–Dawley rats (280–320 g) were randomly divided into sham, standard diet (SD), KD, and BD groups. The sham, SD, and BD groups were fed with *ad libitum* standard food, whereas the KD group was fed with *ad libitum* ketogenic food (Shenzhen Zeneca, Shenzhen, China), in which the ratio of fat to carbohydrate and protein was 4:1. In addition, BD (Macklin, Shanghai, China) was administered daily by gavage for the BD group (1 ml/100 g). The basic nutrients of the ketogenic food and standard food are shown in Table 1.

**TABLE 1:** Basic nutrient content of ketogenic food and standard food (per 100 g).

Items	Ketogenic food*	Standard food
Carbohydrate	8.3 g	55.5 g
Protein	8.3 g	14.5 g
Fat	66.5 g	4 g
Dietary fiber	4.8 g	4.5 g
Calcium	990 mg	720 mg
Phosphorus	880 mg	600 mg
Vitamin D	4.75 μg	2.5 μg
Calorie	664.9 kcal	316 kcal

*The ratio of fat to carbohydrate and protein is 4:1 in classic ketogenic food.

All rats were housed in an artificial environment with 12 h:12 h light/dark circle and had free access to water. All procedures followed the guidance of animal care and were approved by the Ethics Committee for Animal Experiments of Nanfang Hospital, Southern Medical University (Animal ethics no.: NFYY-2019-53).

### Surgical Procedure and C5 Hemi-Contusion Injury

A hemi-contusive C5 SCI model with displacement control was used as described previously (Huang Z. et al., 2018). In brief, rats were anesthetized with 2–3% isoflurane in oxygen via a small animal anesthesia machine (VP1000; Matrix, United States), and injected subcutaneously with 5 ml normal saline to prevent dehydration during the procedure. Then, the rats were fixed to a stereotaxic apparatus (standard type; Ruiwode, China) in a prone position, shaved, and sterilized at the head and neck. A 100 g object was suspended from the tail to stretch the cervical spine. A 2.5-cm median incision in the back starting from C2 was conducted to expose the vertebral plate. After locating the C5 level, the muscle attachments and tissue of C3–C7 were cleaned, and the groove between the corresponding transverse processes and articular processes was exposed. Unilateral laminectomy was performed at the left side of C5 to expose the left side of the spinal cord, while the spinal dura remained intact. A custom clamp was mounted on the C4–C6 groove and then fixed to the stereotaxic apparatus to eliminate spinal cord movement caused by breathing. All surgical instruments underwent autoclave sterilization before operation, and the entire procedure was performed under an operating microscope (YH-X-4A; Zhenjiang Yihuaguang, China).

**FIGURE 1:**
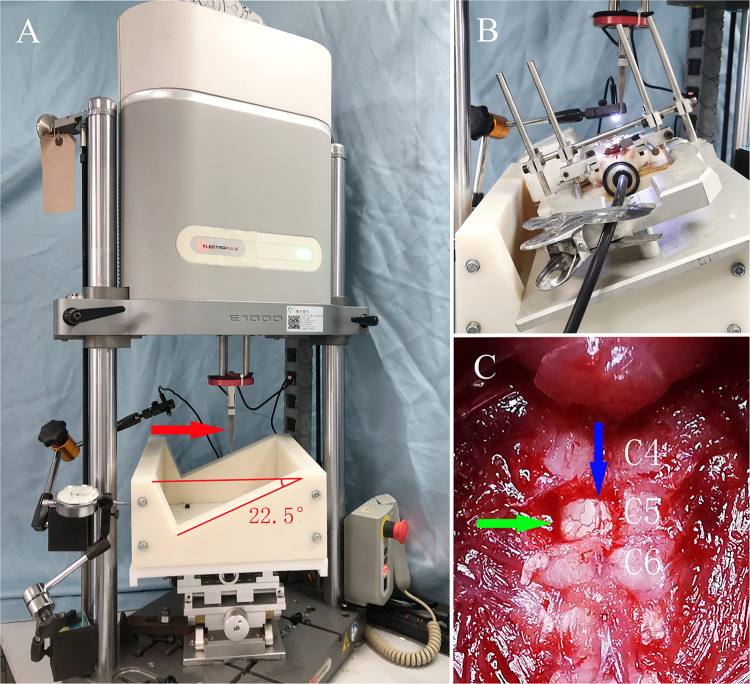
The servo-electromagnetic material testing machine and general view of the SCI model in rats. (**A**) The servo-electromagnetic material testing machine, where the red arrow indicates the impactor (1.5 mm diameter at the tip) connected to a sensor. (**B**) Rat under anesthesia with isoflurane, fixed on a special stereotaxic frame with an angle of 22.5°. (**C**) Exposure of the surgical field, where the green arrow indicates the C5 spinal cord after unilateral laminectomy, while the blue arrow indicates the posterior median vessels of the spinal cord.

After laminectomy, and with a stereotaxic apparatus, the rat was transferred onto a custom frame that was tilted 22.5° and fixed on the x-y-z platform of an electromagnetic servo material testing machine (Instron E1000; Instron, United States) to perform a C5 hemi-contusive SCI (as shown in Figure 1). The 1.5-mm-diameter metal impactor pointed 1.4 mm laterally to the posterior mid-line at the C5 spinal cord. A vibration program was initiated to determine the initial position of the contusion. Then, an impact program was triggered to drive the impactor to contuse the left side of the C5 spinal cord with a set displacement of 1.5 mm at 100 mm/s. The real-time speed, displacement, and force were recorded. After contusion, the muscle and skin were sutured layer by layer, and the incision was sterilized again. Penicillin (10,000 U) was injected intramuscularly to prevent infection. The rats were placed in 37°C incubators until anesthesia recovery and then transferred to the ordinary cage.

For the sham group, unilateral laminectomy was performed without contusion, followed by suture, sterilization, prophylactic anti-infection, and recovery.

### Measurement of Weight, Serum Glucose, and Ketone

The body weight, serum glucose, and ketone level of all rats were measured at pre-injury and at the 1st, 3rd, and 7th day post-injury (dpi). Then, the body weight was monitored on a weekly basis, whereas the serum glucose and ketone levels were recorded every 2 weeks for a period of 12 weeks. Venous blood was collected from the tail vein to detect glucose and ketone with a glucometer (JPS-5; Beijing Yicheng, China) and ketone body tester (T-1; Beijing Yicheng), respectively. Our preliminary experiments showed that serum ketone levels peaked at 6 h after BD gavage, and therefore ketone and glucose detection were conducted at this time point in each group (Supplementary Figure 1).

### Protein and Gene Expression Analyses

At the 7th dpi, eight rats were sacrificed randomly in each group. The spinal cord-containing epicenter was harvested after intracardiac perfusion with 4°C normal saline for gene and protein expression analyses.

#### Gene Expression

Reverse transcription PCR was conducted to analyze the expression of relative genes. Total RNA was extracted from the spinal cord tissue with TRIzol reagent (10296010; Invitrogen, United States). Then, cDNA was synthesized using a Goldenstar RT6 cDNA Synthesis Kit (TSK301S; Beijing Tsingke, China) under the manufacturer’s recommendation. Finally, the 2 × T5 Fast qPCR Mix kit (SYBR Green I) (TSE202; Beijing Tsingke) was used to amplify the gene expression via Quant Studio 5 (A28139; Applied Biosystems, Thermo Fisher Scientific, United States). The sequences of the PCR primers are listed in Table 2.

**TABLE 2:** The oligonucleotide primers used for amplification.

	Sequence
GAPDH	Forward 5′-ACCACCATGGAGAAGGCTGG-3′ Reverse 5′-CTCAGTGTAGCCCAGGATGC-3′
TNF-α	Forward 5′-CCAGGTTCTCTTCAAGGGACAA-3′ Reverse 5′-CTCCTGGTATGAAATGGCAAATC-3′
IL-1β	Forward 5′-CACCTCTCAAGCAGAGCACAG-3′ Reverse 5′-GGGTTCCATGGTGAAGTCAAC-3′
IL-6	Forward 5′-CCAAGACCATCCAACTCATCTTG-3′ Reverse 5′-CACAGTGAGGAATGTCCACAAAC-3′

#### Protein Expression

Proteins were extracted from the spinal cord tissue with a RIPA lysis buffer kit (FD009; FdBio Science, China) and separated in 10 or 12% SDS-PAGE gels (FD2190; FdBio Science). Equal amounts of proteins (30 ng) were added in each lane of the gels. Then, the separated proteins were transferred onto a PVDF membrane (0.45 μm in aperture), followed by blocking with 5% fetal bovine serum albumin (FD0030; FdBio Science) at room temperature for 1 h. The protein bands were incubated at 4°C overnight with the following primary anti-bodies: GAPDH (60004-1-Ig, Proteintech, United States; dilution 1:1000), iNOS (ab49999, Abcam, United Kingdom; dilution 1:1000), Arginase-1 (66129-1-Ig, Proteintech; dilution 1:1000), TNF-α (17590-1-AP, Proteintech; dilution 1:1000), and IL-1β (BS3506, Bioworld, United States; dilution 1:1000). After washing in Tris-buffered saline with 0.1% Tween 20 for 10 min × 3 times, the bands were incubated at room temperature for 1 h with secondary anti-bodies [HRP-conjugated affinipure goat anti-mouse/anti-rabbit IgG (H + L)] (SA00001-1/SA00001-2, Proteintech, United States; dilution 1:1000). Then, the bands were washed with Tris-buffered saline with 0.1% Tween 20 for 5 min × 5 times, and finally visualized with a luminescent imaging workstation (Tanon 6600; Shanghai Tanon, China) after immersing in an ECL chemiluminescent substrate luminescent solution (1705061; Bio-Rad, United States). The images were analyzed with the ImageJ software (v.1.48; Rawak Software, Germany).

### Behavioral Evaluations

**FIGURE 2:**
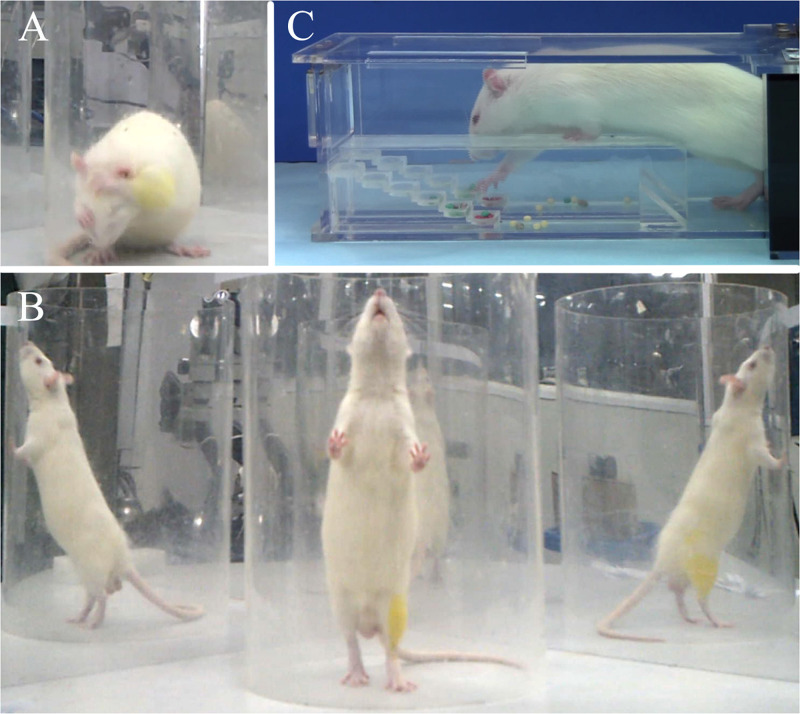
Representative images of behavioral evaluations: (**A**) grooming test, (**B**) cylinder rearing test, (**C**) Montoya’s staircase test.

Behavioral evaluations were conducted at pre-injury and at every 2 weeks post-injury (wpi) for 12 weeks, including grooming test, rearing test, and Montoya’s staircase test. Briefly, the grooming test was used to evaluate the rat’s forelimb function. Cold water was applied on the head and neck to wet the rat’s fur. Then, the rat was placed in a clear Plexiglas cylinder (20 cm diameter, 46 cm height). Two mirrors (50 cm × 60 cm) were placed perpendicularly to each other behind the cylinder, so that the movement details could be clearly assessed from multiple angles. Each rat was videotaped for 15 min. The grooming movement was scored by a non-experimenter by referring to the video frame by frame. Different scores were marked if the forelimbs reached different areas of the face and head: score 1 referred to the area below the lips; score 2 referred to the area between the lips and nose; score 3 referred to the area between the nose and eyes; score 4 referred to the area between the eyes and ears; score 5 referred to movement whereby the forelimbs could flatten the root of the ear in a direction from back to forward. Five sets of independent grooming movements were evaluated for each rat and scored respectively for each side of the forelimbs. The highest score of each side was selected as the final score of that side. The typical grooming movement is shown in Figure 2A.

The rearing test was used to evaluate the forelimbs’ usage. A rat was placed in the cylinder and the mirrors remained in the same position as the grooming test. Each rat was videotaped for 15 min. Twenty independent rearing movements were analyzed by a non-experimenter, again by referring to the video frame by frame. All independent movements were analyzed if there were less than 20 movements in 15 min. Each movement was analyzed as initial rearing (recorded as a = used ipsilateral forelimb only, b = used contralateral forelimb only, c = used both forelimbs) and subsequent wall touch (recorded as d = used ipsilateral forelimb only, e = used contralateral forelimb only, f = used both forelimbs). The usage of each forelimb was calculated as ipsilateral forelimb usage = (a + c + d + f)/(a + b + c + d + e + f) × 100%; contralateral forelimb usage = (b + c + e + f)/(a + b + c + d + e + f) × 100%. The typical rearing movement is shown in Figure 2B.

Montoya’s staircase test was used to evaluate the skill-reaching function of the forelimbs. After grouping, all rats were trained every other day for 2 weeks before modeling, and the result of the final training was used as the baseline. The inducing food was special sugar pills (F0299; Bioserve, United States) with a diameter of 3.5 mm in different colors. The staircase contained six steps, where each one was independent between the left and right sides. Each step contained a shallow well, filled with four sugar pills. The rat could only enter the staircase from one side, and the left or right forelimb could only grasp the food on the left or right steps, respectively. Rats were fasted for 12 h before the test to enhance motivation. In the formal experiment, rats were placed in the staircase for 15 min under a dark and quiet environment. The exact number and position of the remanent sugar pills were recorded at the end for both sides. The typical food grasping movement is shown in Figure 2C.

### H&E Staining

At 12 wpi, the rats were sacrificed by intracardiac perfusion with 0.1 M PBS (300 ml) and 4% paraformaldehyde (4°C, 300 ml) in deep anesthesia. A 6 cm length of spinal cord containing the epicenter was collected from each rat and then post-fixed in 4% paraformaldehyde; cryoprotected in 12, 18, and 24% sucrose solution; embedded with a freezing embedding agent (4583; Sakura, United States); and sectioned (20 μm per cut) consecutively onto slides at its axial plane with a cryotome (FSE; Thermo Scientific, United States).

H&E staining was conducted to visualize the lesion area, and the spared white and gray matter. In brief, frozen spinal cord sections were dried at 50°C for 1 h, then rinsed in 0.01M PBS solution for 3 min, and stained in hematoxylin solution for 10 min. After rinsing in dH_2_O twice, the slides were differentiated in 1% ethanol HCl for several seconds, blued in a sodium bicarbonate solution for 30 min, and then stained in eosin solution for 2 min, before finally being cover-slipped with a Permount mounting medium. The slides were photographed (BX63; Olympus, Japan), and the epicenter of the contusion was analyzed with the ImageJ software. The spared ipsilateral white and gray matter were standardized contralaterally, whereas the lesion area was standardized by the total area of the cross-section.

### Statistical Analyses

GraphPad Prism (v.8.0.1; GraphPad Software, United States) was used for image processing, whereas Statistica (v.7.1.216.0; StatSoft, United States) was used for the statistical analyses. The data of the peak force, gene and protein expression, and histology evaluation were analyzed by one-way ANOVA, with the data of the general indicators and behavioral evaluation analyzed by two-way ANOVA. The Student–Newman–Keuls test was used for multiple comparisons between groups. All results were expressed as the mean ± SD. Statistical significance was established at *p* < 0.05.

## Results

### The Biomechanical Parameters of the SCI Model Were Consistent Among the Injury Groups

The real-time displacement, speed, and force during the SCI molding process were recorded, with the actual impact displacement, maximum speed, and peak force summarized in Table 3. Compared with the average displacement of all samples, the variation of the SD, KD, and BD groups were all lower than 0.33%. Compared with the average maximum speed of all samples, the variation of the SD, KD, and BD groups were 0.54, 1.19, and 0.71%, respectively. No significant difference was observed in peak force between groups (*p* = 0.2670), indicating good consistency in SCI severity between the three groups.

**TABLE 3:** The biomechanical parameters of the spinal cord contusion model.

	Displacement (mm)	Maximum speed (mm/s)	Peak force (N)*
SD	1.502 ± 0.022	97.215 ± 1.999	0.797 ± 0.169
KD	1.507 ± 0.038	98.913 ± 2.149	0.701 ± 0.140
BD	1.497 ± 0.039	97.052 ± 2.336	0.783 ± 0.143
Average	1.502 ± 0.034	97.747 ± 2.303	0.757 ± 0.150

*No significant difference was observed in peak force among groups (*p* = 0.2670, one-way ANOVA).

*All data are expressed as mean ± SD.*

### Change in Weight and Serum Glucose

**FIGURE 3:**
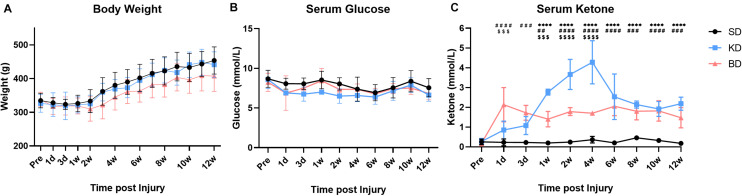
The basic data of the rats at pre-injury and post-SCI in 12 weeks. Significant difference was found in weight, serum glucose, and ketone, respectively (two-way ANOVA). (**A**) The weight of the BD group was lower than the KD and SD groups (Student–Newman–Keuls test). (**B**,**C**) The KD group presented a slightly lower serum glucose level, but the highest ketone level among the three groups (Student–Newman–Keuls test). The serum ketone of the BD group stabilized at a certain level and was higher than the SD group (Student–Newman–Keuls test). Symbols *, #, and $ indicate significant difference at each time point in SD vs. KD, SD vs. BD, and KD vs. BD, respectively (Student–Newman–Keuls test). One, two, three, and four symbols indicate *p* < 0.05, *p* < 0.01, *p* < 0.001, and *p* < 0.0001, respectively.

The variation trend of body weight and serum glucose level in the early stage and 12 weeks post-SCI are presented in Figures 3A,B. The weight tended to decrease slightly at the first 1 or 2 weeks after injury, and then to recover and increase gradually. Multiple comparisons between the groups indicated that the weight of the BD group was lower than the KD and SD groups (both *p* < 0.0001), whereas no significant difference was observed between the KD and SD groups. However, there was no significant difference between the groups at each time point. The serum glucose levels in the SD group were significantly higher than the KD (*p* < 0.001) and the BD groups (*p* < 0.01) were lower than the SD group, whereas no significant difference was observed between the KD and BD groups. The serum glucose fluctuated at a similar level (range: 6.5–8.5 mmol/L), but no significant difference was found between groups at every single time point.

### KD and BD Promoted the Serum Ketone Level With Different Patterns After Intervention

As shown in Figure 3C, the order of serum ketone level in the injury groups was KD > BD > SD (all *p* < 0.0001). The KD and BD groups promoted the serum ketone level with different patterns post-intervention. At the early stage (1 dpi), the ketone level of the BD group (2.13 ± 0.87 mmol/L) was significantly higher than the KD (0.85 ± 0.45 mmol/L; *p* < 0.0001) and the SD (0.23 ± 0.17 mmol/L; *p* = 0.0003) groups. At 3 dpi, although the ketone level of the BD group (1.72 ± 0.38 mmol/L) tended to decrease slightly, it was still higher than the SD group (0.23 ± 0.10 mmol/L; *p* < 0.0001). The ketone level of the KD group (1.08 ± 0.46 mmol/L) tended to increase, and the difference between the KD and BD groups disappeared. At 7 dpi, the ketone level of the KD group (2.77 ± 0.15 mmol/L) was significantly higher than the BD (1.40 ± 0.39 mmol/L; *p* = 0.0001) and the SD (0.20 ± 0.10 mmol/L; *p* < 0.0001) groups. Meanwhile, the ketone level of the BD group was still higher than the SD group (*p* = 0.0015). The ketone level of the KD group kept increasing at 1 wpi, peaked at 4.28 ± 1.09 mmol/L at 4 wpi, and then decreased and stabilized at approximately 2.2 mmol/L from 6 to 12 wpi. The ketone level of the BD group was quite stable in the long term, where it reached approximately 2.0 mmol/L at the early stage and fluctuated at this level through 12 weeks. Serum ketone in the SD group maintained at the basal level of 0.2–0.3 mmol/L at each measure times.

### KD and BD Inhibited the Gene Expression of TNF-α and IL-6

**FIGURE 4:**
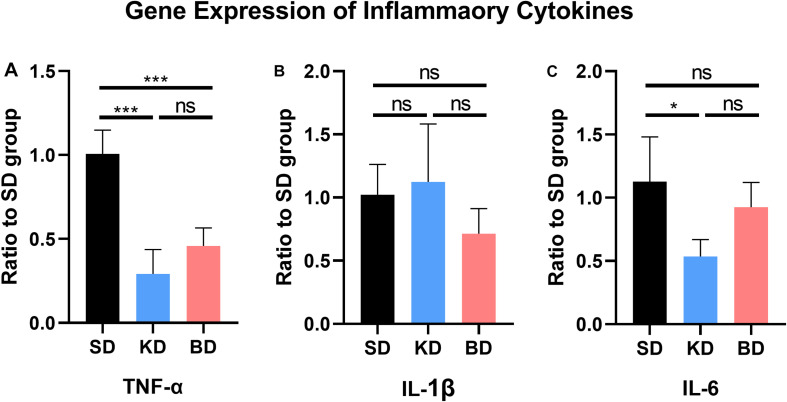
Gene expression of inflammatory factors at 7 dpi. **(A)** TNF-α, (**B**) IL-1β, (**C**) IL-6. Significant difference was observed in the expression of TNF-α and IL-6 (one-way ANOVA). KD and BD significantly inhibited the gene expression of TNF-α, while the IL-6 expression was also significantly inhibited in the KD group (Student–Newman–Keuls test). Symbol * indicates significant difference between groups. One, two, and three symbols indicate *p* < 0.05, *p* < 0.01, and *p* < 0.001, respectively, while ns indicates no statistical difference between groups.

The gene expression of inflammatory cytokines from the SD group were used as the control group. As shown in Figure 4, compared with the SD group, the gene expression of TNF-α from the KD (*p* = 0.0004) and BD (*p* = 0.0006) groups were inhibited, whereas no significant difference was observed between the KD and BD groups. There was no significant difference in the gene expression of IL-1β among the three groups. The gene expression level of IL-6 in the KD group was significantly lower than the SD group (*p* = 0.0254). Although the average IL-6 expression of the BD group tended to be lower than the SD group, no statistical significance was found between these groups.

### KD and BD Inhibited the Protein Expression of iNOS While Promoting the Expression Arginase-1

**FIGURE 5:**
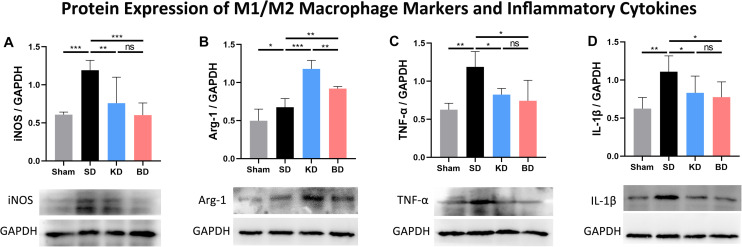
Protein expression of M1/M2 macrophage markers and inflammatory factors at 7 dpi. (**A**) iNOS/GAPDH, (**B**) Arg-1/GAPDH, (**C**) TNF-α/GAPDH, (**D**) IL-1β/GAPDH. Significant difference was observed in the protein expression of all indicators (one-way ANOVA). KD and BD significantly inhibited the protein expression of iNOS, while promoting the expression of Arg-1 (Student–Newman–Keuls test). TNF-α and IL-1β expression were also inhibited in both the KD and BD groups (Student–Newman–Keuls test). Symbol * indicates significant difference between groups. One, two, and three symbols indicate *p* < 0.05, *p* < 0.01, and *p* < 0.001, respectively, while ns indicates no statistical difference between groups.

As illustrated in Figure 5, the protein expression of each group was standardized by the corresponding expression of reduced glyceraldehyde-phosphate dehydrogenase (GAPDH). Inducible nitric oxide synthase (iNOS) and arginase-1 (Arg-1) were typical membrane markers of the M1 and M2 macrophage, respectively. Compared with the sham group, the protein expression of iNOS in all injury groups tended to increase, and only the difference between the SD and sham groups was significant (*p* = 0.0003). When compared with the SD group, the iNOS expression was significantly decreased in the KD (*p* = 0.0015) and BD (*p* = 0.0004) groups. No significant difference was observed between the KD and BD groups, either between these groups or the sham group. Compared with the sham group, the protein expression of Arg-1 in the other groups significantly increased (sham vs. SD, *p* = 0.0231; sham vs. KD, *p* = 0.0002; sham vs. BD, *p* = 0.0002). Compared with the SD group, significantly more Arg-1 expression was detected in the KD (*p* = 0.0002) and BD (*p* = 0.0033) groups; meanwhile, the KD group presented higher Arg-1 expression level than the BD group (*p* = 0.0023).

### KD and BD Inhibited the Protein Expression of TNF-α and IL-1β

The protein expression of TNF-α in the three injury groups tended to be higher than the sham group, and only the difference between the SD and sham group was significant (*p* = 0.0038). Compared with the SD group, TNF-α expression was significantly suppressed in the KD (*p* = 0.0138) and BD (*p* = 0.0106) groups when compared with the SD group, whereas there was no statistical difference between the KD and BD groups. The three injury groups tended to present higher IL-1β protein expression than the sham group, and only the difference between the SD and sham group was significant (*p* = 0.0060). Compared with the SD group, the protein expression level of IL-1β was significantly lower in the KD (*p* = 0.0390) and BD (*p* = 0.0394) groups, and no significant difference was observed between the two intervention groups.

### Rats in the KD Group Presented Higher Usage of Ipsilateral Forelimb in the Rearing Test

**FIGURE 6:**
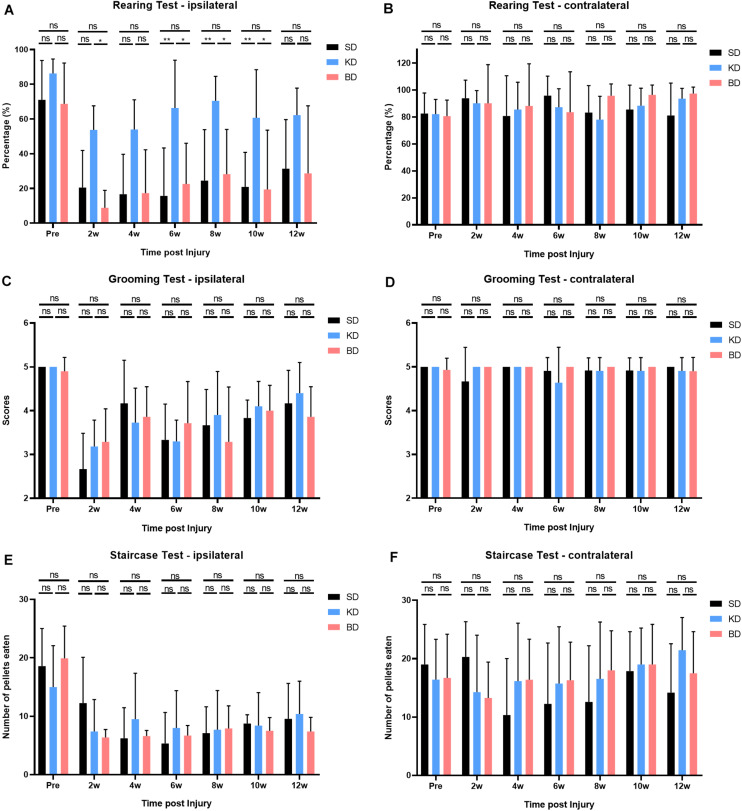
Behavioral evaluations after SCI in 12 weeks. (**A**) Significant difference was observed in the ipsilateral rearing test (two-way ANOVA). Further analyses showed that the KD group presented a higher usage of the ipsilateral forelimb than the SD and BD groups, and no significant difference was found between the BD and SD groups (Student–Newman–Keuls test). (**C**,**E**) No significant difference was observed in ipsilateral grooming and staircase tests, (**B**,**D**,**F**) nor in all the contralateral behavioral tests (two-way ANOVA). Symbol * indicates significant difference between groups. One, two, and three symbols indicate *p* < 0.05, *p* < 0.01, and *p* < 0.001, respectively, while ns indicates no statistical difference between groups.

The results of rearing, grooming, and staircase tests are shown in Figure 6. Both the group factor and time factor had a significant effect on the rearing test (both *p* < 0.0001), without interaction effect. Compared with the pre-injury, the usage of the ipsilateral forelimb decreased in all injury groups post-SCI, and there was significant difference among the groups (*p* < 0.0001). Multiple comparisons indicated that the usage of the forelimb in the KD group was significantly higher than the SD and BD groups (*p* < 0.0001). No statistical difference was observed between the SD and BD groups at all time points.

The time factor had a significant effect on the grooming test (*p* < 0.0001), whereas the group factor did not, and there was no interaction effect between the two factors. Compared with the pre-injury, the grooming scores of the ipsilateral forelimb decreased in all groups after SCI. However, no significant difference among the groups was presented, and the scores tended to increase by time post-injury.

The time factor had a significant effect on the staircase test (*p* < 0.0001), whereas the group factor did not, and there was no interaction effect between the two factors. Compared with the pre-injury, the consumption of sugar pills via the ipsilateral forelimb decreased in all groups after SCI. No significant difference among the groups was observed, and the consumption did not tend to increase by time after injury. However, during the middle and late stage post-SCI (i.e., from 4 to 12 wpi), the consumption in the KD and BD groups tended to be slightly higher than the SD group and tended to increase by time.

### Rats in the KD Group Preserved a Greater Spared Gray Matter in Histological Evaluation

**FIGURE 7:**
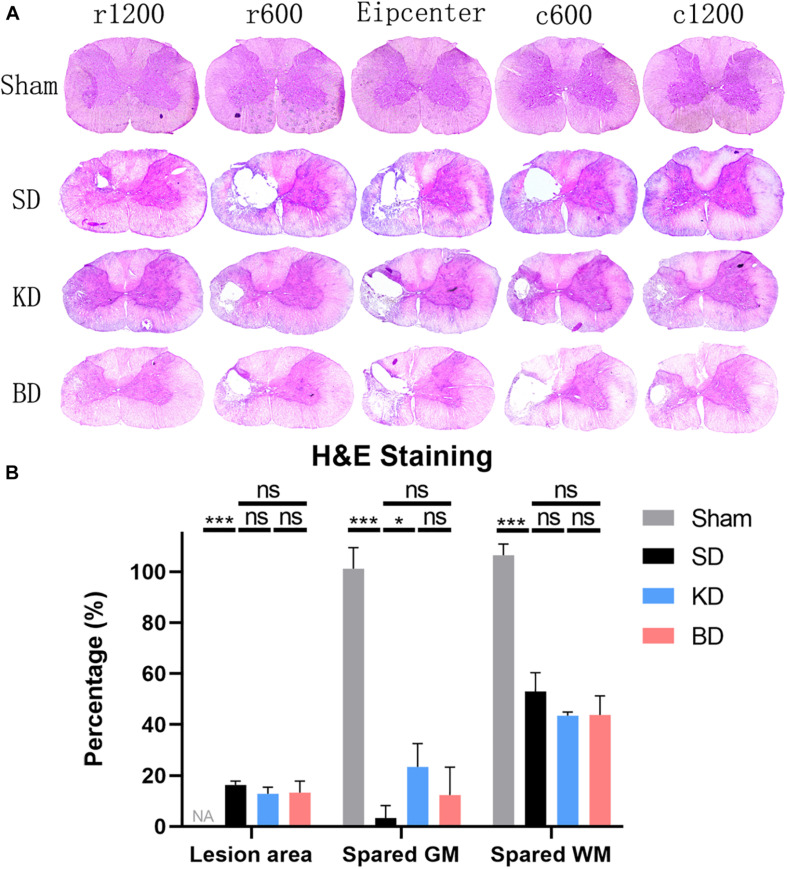
Representative H&E staining images at 12 wpi. (**A**) The epicenter and 600 μm, 1200 μm rostral and caudal to the epicenter of the SCI in the three groups. All three injury groups showed an obvious vacant area. (**B**) H&E histological quantification of the epicenter, where both the KD and BD groups tended to present higher areas of spared gray matter than the SD group, but significant difference was only found between the KD group and the SD group (by Student–Newman–Keuls test). Symbol * indicates significant difference between groups. One, two, and three symbols indicate *p* < 0.05, *p* < 0.01, and *p* < 0.001, respectively, while ns indicates no statistical difference between groups. GM, gray matter; WM, white matter.

Representative H&E staining images of the C5 spinal cord cross-sections are shown in Figure 7A. The longitudinal damage, presented by rostral–epicenter–caudal lesion area, exceeded the diameter of the impactor (1.5 mm). The longitudinal damage in the KD and BD groups tended to be less severe than the SD group. The spared white and gray matter and lesion area were measured and standardized, and are illustrated in Figure 7B. Rats in the KD group presented a significantly greater spared gray matter than those in the SD group (*p* = 0.0192). The mean spared gray matter of the BD group tended to be greater than the SD group, although the difference was not significant. There was no significant difference in spared white matter among the groups or in the lesion area.

## Discussion

The present study investigated the neuroprotection of ketone metabolism, including KD and BD, after cervical spinal cord hemi-contusion in rats. The results indicated that ketone metabolism induced by KD presented benefit in terms of the improvement of motor function and the reservation of spinal gray matter after SCI. Such neuroprotective effect might be related to regulation of the inflammatory response and to the inhibition of M1 and promotion of M2 macrophage polarization. However, ketone metabolism induced by BD regulated the macrophage polarization and inflammatory response without motor function improvement.

A hemi-contusive cervical SCI model with displacement control was used in the present study. The displacement and speed were the set parameters, and the maximum variation of actual displacement and speed was less than 1.2%. As an unset parameter, the peak force showed no significant difference between the injury groups, suggesting that the severity of injury in this model was highly consistent and thus applicable for subsequent intervention.

At the early stage post-SCI, the weight of the animals temporarily dropped because of a sharp decrease in exercise and food intake. After adapting to the pathological state, the weight gradually recovered as food consumption increased. In addition, the satiety caused by gavage in the BD group also led to a decrease in food intake, so that the weight in the BD group was slightly lighter than the others. Compared with the SD group, the KD and BD groups did not present apparent hypoglycemia. However, the serum glucose of the KD and BD groups was slightly lower than the SD group, and the KD group was the lowest. This was probably because the unlimited supply of ketogenic food resulted in a more complete ketone metabolism in the KD group, and thus the glycometabolism was reduced. Because of the process of gluconeogenesis in the body, however, no obvious hypoglycemia was observed. This phenomenon is consistent with the results of our previous clinical trial (Guo et al., 2014). The changes of serum ketone in the two treatment groups were different. After gavage of BD, the serum ketone in the BD group reached a stable level in a short time, and plateaued earlier than the KD group. The serum ketone in the KD group rose slowly, but peaked at a higher value, and then decreased and plateaued at a similar level to the BD group. This phenomenon likely resulted from the different mechanism of intervention. For the BD group, the frequency of gavage was once a day, without interruption of normal diet. There was no timely supplement after the BD was absorbed and metabolized in the liver, and thus the ketone level in the systemic circulation was limited. However, the management of BD gavage ensured the ketone level during the first few days post-SCI. This was of significance in the treatment of SCI as an early intervention. As for the KD group, standard food was replaced with ketogenic food, without intake limitation. Therefore, serum ketone accumulated gradually after metabolism, and then re-balanced and fluctuated at a certain level when the animal accommodated the ketone metabolism. The changes in ketone levels caused by ketogenic food and BD were inconsistent at the early stage, but similar at the middle and late stages of intervention. Therefore, it was necessary to compare the effect of this difference on SCI.

The inflammatory response, in which macrophage is crucial for the process, is one of the important characteristics of secondary SCI. Under the effect of chemokines, monocyte migrates into the spinal cord from circulation and differentiates into macrophage. Then, the macrophage begins to gather in and around the injury site at 3 dpi. The number of macrophages peaks at 7 dpi, and then slightly decreases, before it begins to rise again at 14 dpi, and peaks a second time at 60 dpi (Milich et al., 2019). Previous study indicated that facilitating the polarization of macrophage into the M2 sub-type had a beneficial effect on the repair of central nervous system injury (Kigerl et al., 2009). Moreover, *in vitro* and *in vivo* studies indicated that the ketone body metabolite, βOHB, could activate the differentiation of microglia into the M2 sub-type, exert an anti-inflammation effect in the nervous system, and reduce depression-like behavior (Huang C. et al., 2018). The results of the present study showed that compared with the control group, the KD and BD effectively reduced the expression of iNOS, one of protein markers of M1 macrophage, while promoting the expression of Arg-1, one of the protein markers of M2 macrophage. It is suggested that ketone metabolism induced by these two methods could affect the polarization process of microglia/macrophage in spinal cord tissue. To be specific, the ketone metabolism inhibits the M1 and enhances M2 macrophage polarization.

Inflammatory cytokine can directly reflect the inflammatory response after SCI. TNF-α and IL-1β are commonly used to simulate *in vitro* aseptic inflammation to induce the polarization of M1 macrophage. Both factors are highly expressed in the spinal cord post-trauma. The results of the present study suggest that ketone metabolism tended to reduce the expression of inflammatory cytokines after SCI. The gene and protein expression of TNF-α were suppressed significantly. The protein expression of IL-1β and gene expression of IL-6 also tended to be inhibited in rats of both the KD and BD groups, indicating that ketone metabolism can regulate the inflammatory response in spinal cord tissue.

Our results also indicate that KD improved the motor function following acute SCI. The rearing and grooming tests reflected the motor function of the large joint of forelimbs in rats, while the Montoya’s staircase test reflected the skilled reaching function of the fore paws. The rats fed with ketogenic food had a higher usage of the ipsilateral forelimb in the rearing test than rats from other groups after SCI. However, no apparent improvement on skilled reaching presented in the KD group, indicating that ketone metabolism had a more significant effect on the recovery of large joint movement after SCI in rats. There was no improvement on the behavioral evaluations in rats gavaged with BD, possibly because the serum ketone level of the BD group did not rise as high as the KD group in the first few weeks post-SCI, which is a crucial period in which to conduct efficient treatments to improve serum ketone to a certain level. On the other hand, it might be because the gavage imposed a negative influence on behaviors, leaving less enthusiasm for movement in rats. Interestingly, the study of Streijger et al. (2013) showed that KD was effective in improving both the motor function of large joints and skilled reaching, whereas the KD only benefited the former in our study, although this might be due to the different contusion SCI models in the two studies, where Streijger et al. (2013) used the force-controlled contusion model with an Infinite Horizon impactor, while a displacement-controlled contusion model was adopted in our study. These two models might result in inconsistent severity of injury and have different effects after the treatment intervention.

It was indicated in the present study that KD increased the spared gray matter at the spinal cord epicenter when compared with the control group. However, the rats of the BD group did not show such results. Moreover, the beneficial effect of ketone metabolism induced by the two methods was limited in reducing the lesion area at the epicenter, as well as in reserving the spared white matter at the ipsilateral side. The gray matter of the spinal cord is the site where neurons gather, and the survival of neurons is critical for the improvement of motor function after SCI. The neuroprotective effect of ketone metabolism has been verified in the field of neuroscience, with Cheng et al. (2009) finding that KD could resist the neurotoxicity of 6-hydroxydopamine and protect dopaminergic neurons in substantia nigra in the Parkinson’s disease model. Kashiwaya et al. (2000) proposed that during the *in vitro* culture of midbrain neurons and hippocampal neurons, the addition of 4 mM d-gab-hydroxybutyrate could protect neurons from the toxic effects of heroin analogs (MPP1) and amyloid protein (Aβ_1–42_), which are important risk factors in Parkinson’s disease and Alzheimer’s disease, respectively. In terms of SCI, Guo et al. (2015) observed that musk ketone reduced the neuronal degeneration and necrosis in spinal cord tissue at 7 dpi in rats. In our study, the result of the behavioral evaluation in the KD group was superior to the other two groups, whereas it was consistent with the result of histological evaluation in which the KD group presented with greater spared gray matter. These results support the view that KD-induced ketone metabolism has a protective effect on neurons.

There were limitations in this study. First, no behavioral improvement was observed in the BD gavage group, as a gradient dose of BD was not considered in the present study. We suppose that a higher dose of BD could promote the behavioral outcomes. Thus, the optimal dose of BD, especially at the early stage of SCI, should be addressed in future study. Second, although the iNOS and Arg-1 are primarily expressed in M1 and M2 macrophage, respectively, they could also exist in other cells of the central nerve systems and be upregulated by spinal cord pathology. For example, Arg-1 was detected in both macrophage and reactive astrocytes after SCI (Ahn et al., 2012), whereas iNOS was expressed in microglial cells, astrocytes, and neurons, especially in experimental autoimmune encephalomyelitis models (Sonar and Lal, 2019). Therefore, the multiple cell types expressing iNOS and Arg-1 may interfere with the interpretation of the Western blot results.

## Conclusion

The present study indicates that both KD and BD effectively increase the serum ketone level post-SCI in rats, in which the effect works earlier in BD, while being stronger and longer lasting in KD. Both treatments inhibit the expression of inflammatory cytokines and may regulate the macrophage polarization to the M2 sub-type. Moreover, ketone metabolism induced by KD, rather than by BD, seems to have a beneficial effect on the improvement of motor function and on the reservation of spared gray matter after SCI.

## Data Availability Statement

The raw data supporting the conclusions of this article will be made available by the authors, without undue reservation.

## Ethics Statement

The animal study was reviewed and approved by The Ethics Committee for Animal Experiments of Nanfang Hospital, Southern Medical University.

## Author Contributions

XLW and QZ conceived and supervised the study. XLW, JYL, and ZCH designed the experiments. JYL, ZCH, JHL, ZPH, YL, QL, ZY, RL, XHW, and ZS performed the experiments. JYL, ZPH, and RL contributed to animal management. XLW, QAZ, and JYL analyzed the data. JYL wrote the manuscript. All the authors read and approved the manuscript.

## Conflict of Interest

The authors declare that the research was conducted in the absence of any commercial or financial relationships that could be construed as a potential conflict of interest.

## Supplementary Material

Supplementary Figure 1 The change of ketone level after BD gavage by time. The serum ketone level of the BD group was significantly higher than the SD group at all time points (by Student–Newman–Keuls test) after the administration of BD, and the peak value occurred at the 6th hour post-gavage. Thus, the detection of serum ketone in the formal experiment was conducted at this time point.

## Abbreviations

β OHB: beta-hydroxybutyrate
Arg-1: arginase-1
BD, 1: 3-butanediol
dpi: days post-injury
GAPDH: glyceraldehyde-phosphate dehydrogenase
GM: gray matter
iNOS: inducible nitric oxide synthase
KD: ketogenic diet
SCI: spinal cord injury
SD: standard diet
WM: white matter
wpi: weeks post-injury.
