# Supplementary figures and images for: Neuroprotective Effect of Ketone Metabolism on Inhibiting Inflammatory Response by Regulating Macrophage Polarization After Acute Cervical Spinal Cord Injury in Rats
Source: Front Neurosci. 2020 Oct 23;14:583611. doi: 10.3389/fnins.2020.583611 (PMC7645058; doi:10.3389/fnins.2020.583611)

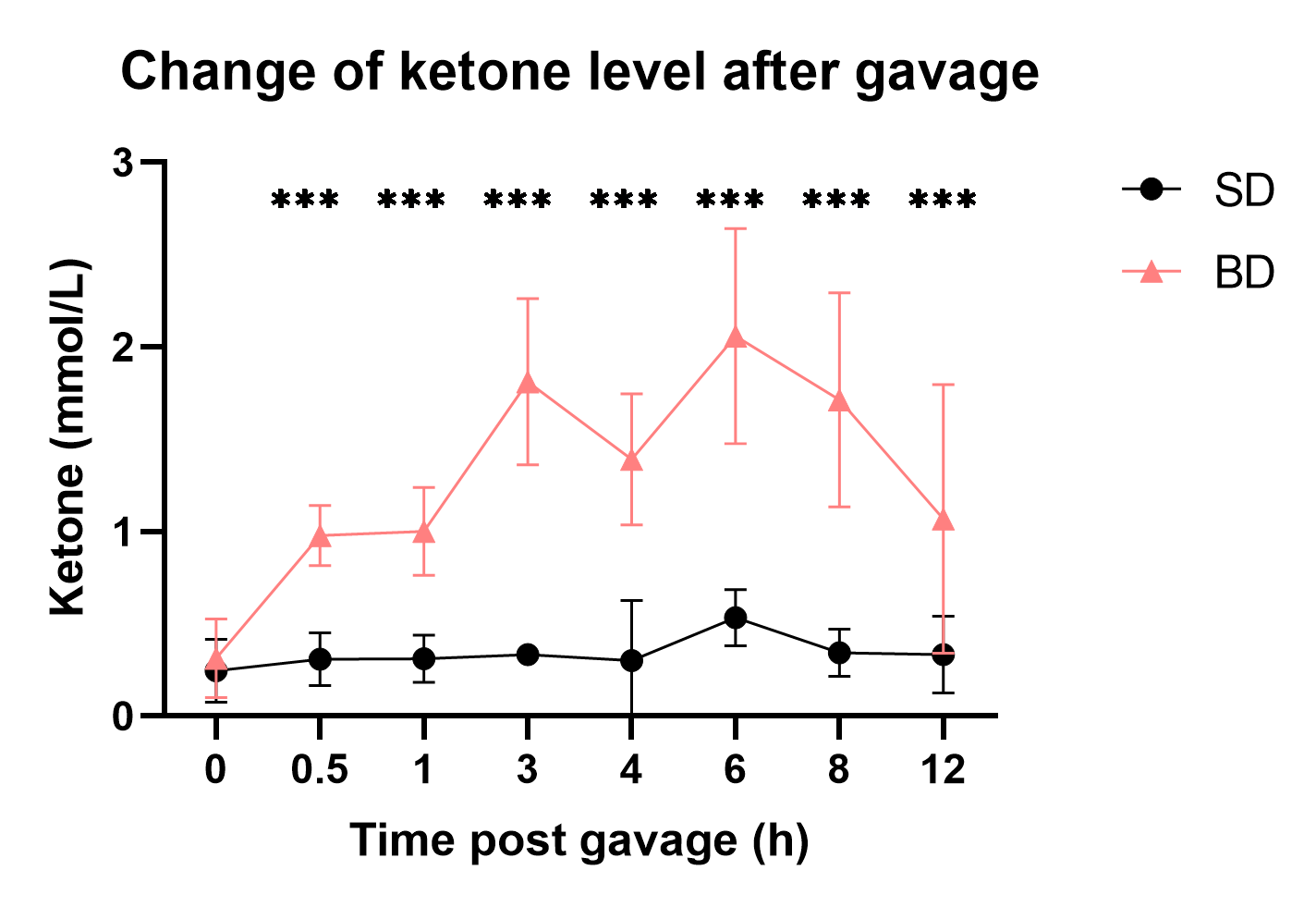

Supplement: Supplementary Figure 1 — The change of ketone level after BD gavage by time. The serum ketone level of the BD group was significantly higher than the SD group at all time points (by Student–Newman–Keuls test) after the administration of BD, and the peak value occurred at the 6th hour post-gavage. Thus, the detection of serum ketone in the formal experiment was conducted at this time point. [file Image_1.TIF]
